# Interplay between trauma and *Pseudomonas entomophila* infection in flies: a central role of the JNK pathway and of CrebA

**DOI:** 10.1038/s41598-017-14969-7

**Published:** 2017-11-24

**Authors:** Ramy Ragheb, Alexandre Chuyen, Magali Torres, Arnaud Defaye, Denis Seyres, Laurent Kremmer, Nicolas Fernandez-Nunez, Hervé Tricoire, Pascal Rihet, Catherine Nguyen, Laurence Röder, Laurent Perrin

**Affiliations:** 1 Inserm UMR_S 1090, TAGC, parc scientifique de Luminy, case 908, 13288 Marseille cedex 9, France; 2 Aix-Marseille université, TAGC, parc scientifique de Luminy, 13288 Marseille, France; 3 Université Paris Diderot-Paris 7 /CNRS, UMR8251, 4, rue Marie Andree Lagroua Weill Halle, 75013 Paris, cedex 13 France; 4 CNRS, Marseille, France

## Abstract

In mammals, both sterile wounding and infection induce inflammation and activate the innate immune system, and the combination of both challenges may lead to severe health defects, revealing the importance of the balance between the intensity and resolution of the inflammatory response for the organism’s fitness. Underlying mechanisms remain however elusive. Using Drosophila, we show that, upon infection with the entomopathogenic bacterium *Pseudomonas entomophila (Pe)*, a sterile wounding induces a reduced resistance and increased host mortality. To identify the molecular mechanisms underlying the susceptibility of wounded flies to bacterial infection, we analyzed the very first steps of the process by comparing the transcriptome landscape of infected (simple hit flies, SH), wounded and infected (double hit flies, DH) and wounded (control) flies. We observed that overexpressed genes in DH flies compared to SH ones are significantly enriched in genes related to stress, including members of the JNK pathway. We demonstrated that the JNK pathway plays a central role in the DH phenotype by manipulating the Jra/dJun activity. Moreover, the CrebA/Creb3-like transcription factor (TF) and its targets were up-regulated in SH flies and we show that CrebA is required for mounting an appropriate immune response. Drosophila thus appears as a relevant model to investigate interactions between trauma and infection and allows to unravel key pathways involved.

## Introduction

In mammals, infections trigger an inflammatory response, which activates the innate immune system. The host response to infection occurs through PAMP (Pathogens Associated Molecular Patterns), which are agonists for host receptors, such as Toll like receptors (TLRs). It has recently been recognized that the same pathways are also able to respond to host molecules DAMP (Danger-Associated Molecular Pattern), which accumulate during cell injury and death, leading to the inflammatory response consecutive to tissue injury and the innate immune system activation^1^. Indeed, the initial host response to infection is very similar to that of sterile inflammation consecutive to severe trauma, burn, as well as reperfusion of ischaemic tissues or other forms of tissue damages that lead to cell necrosis^2^.

Importantly, the balance between the intensity and resolution of the inflammatory response is central for the organism’s fitness. For instance, morbidity and mortality due to sepsis is likely caused by an excessive host pro-inflammatory response to an infection in the first few days^3,4^. Since infection and sterile inflammation share common cellular and molecular pathways, it is not surprising that the occurrence of both a sterile injury and an infection can lead to increased risks of sepsis in humans^5^. Indeed, combining both threats may increase the risk of hyper-inflammatory response. However, the mechanisms that are involved in this conflicting interplay between trauma and infection, which culminate in an uncontrolled inflammatory response, remain unknown.

Innate immunity has emerged as the defensive frontline that coordinates the responses to both injury and infection and is evolutionary conserved. Pioneering work in Drosophila has paved the way for the identification of TLRs and other pathways central for the innate immune response following infection in animals^6^. Recent advances pointed out that Drosophila are also able to mount an inflammatory response to wounding^7,8^. In flies, sterile inflammation involves the recruitment of circulating immune cells, which are able to clear debris^9^, and the activation of an humoral response, which involves innate immune pathways and culminates in the secretion of antimicrobial peptides (AMP)^10^. Importantly, it has been demonstrated that uncontrolled systemic wound response is detrimental to flies survival^11^ and previous work established that sterile wounding can have adverse effects on flies survival to infection^12^.

An important issue is to identify the molecular pathways, which are involved in the very first steps of the systemic response to hyper-inflammation. In this study, we set up a robust assay to analyze interactions between trauma and septic injury in Drosophila. We show that flies subjected to both a sterile trauma and a septic injury using *Pseudomonas entomophila* (*Pe*; double hit flies, DH) are less resistant to an infection compared to flies subjected to septic injury alone (simple hit flies, SH), suggesting that, in flies also, acute inflammation has adverse effect on the ability to fight this pathogen. To investigate the molecular pathways involved, we further compared the transcriptome landscapes of DH, SH and control (only wounded) flies. This pointed out a delayed immune response and an exacerbated stress response in DH flies. In particular, the JNK pathway was up-regulated in DH flies. By manipulating Jra/dJun activity in adult flies, we provide evidence that the JNK pathway activation is instrumental in the sensitivity to infection of DH flies. In addition, the CrebA/Creb3-like transcription factor (TF) and its targets were up-regulated in SH flies and we show that CrebA is required for mounting an appropriate innate immune response. Drosophila thus appears as a relevant model to investigate the complex interactions between inflammation and infection and allows to unravel key pathways involved in the development of a hyper-inflammatory state.

## Results

### Wounding reduces survival upon infection in flies

**Figure 1:**
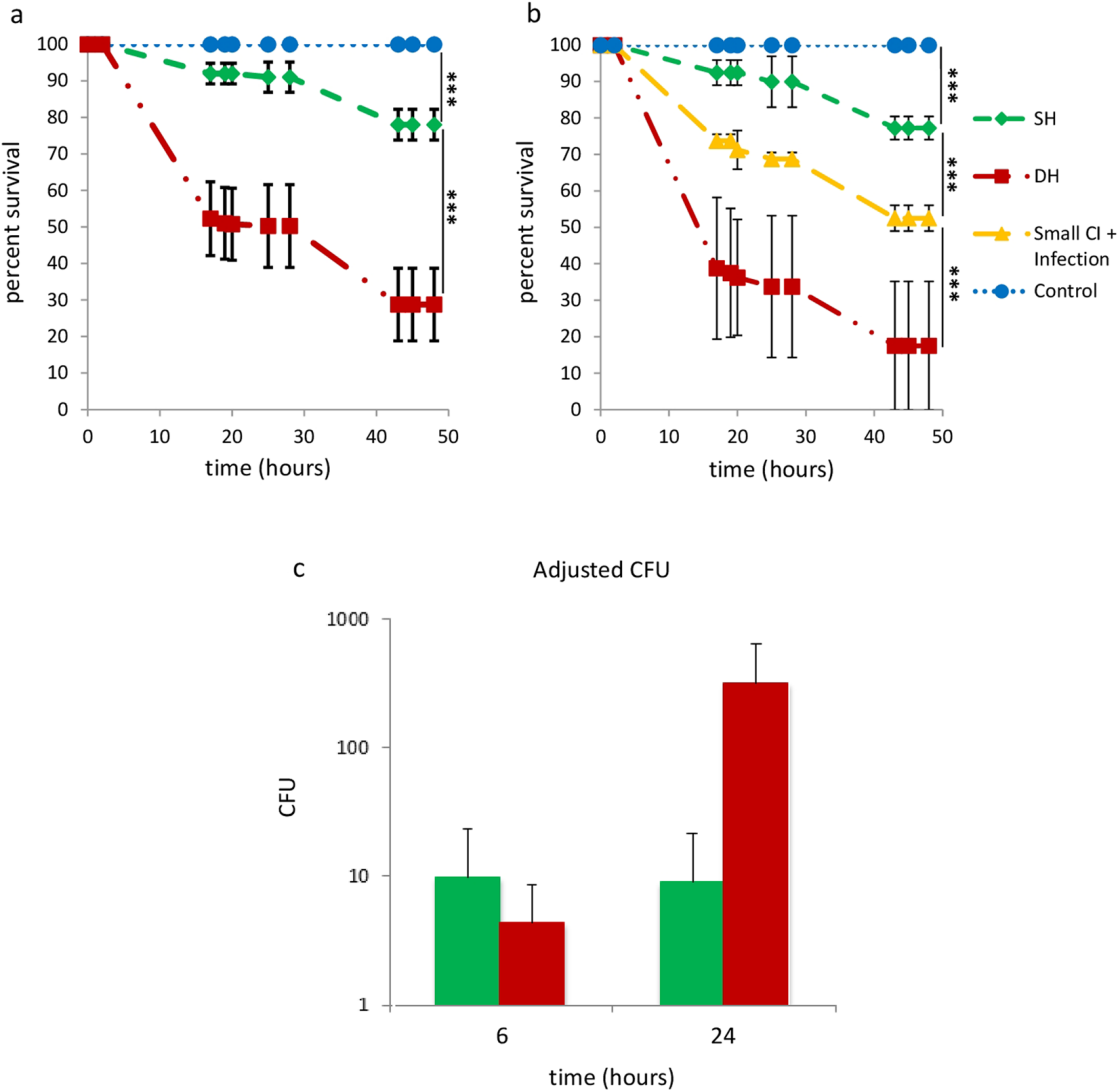
Wounding reduces flies resistance to infection. (**a**) Effect of trauma on survival rate of wild-type infected flies. Survival of flies subjected to both sterile wounding and infection (red, DH) was severely compromised compared to that of flies subjected to infection only (green, SH) and that of control sham flies subjected to sterile infection only (blue, Control). Mean and standard deviations of survival based on 10 experiments each performed on 40 flies per condition is shown. Time 0 is the time at which trauma and infection were performed. (**b**) Effect of wounding size on the survival rate of DH flies. Red: DH flies. Yellow: DH flies injured with a small clean needle (small CI). Mean of 2 experiments each performed on 40 flies per condition. Time 0 is the time at which trauma and infection were performed. The more the wound was large, the more the survival rate was reduced. (**c**) SH (green), DH (red) and control sham flies were analyzed for bacterial load at 0, 6 and 24 hours post-experiment. For each time point, 3 flies per group were analyzed, and two independent experiments were performed. CFU: Colony Forming Unit. Crude CFU was adjusted at 6 and 24 hours by dividing CFU at these times by CFU at 0 hour. Thus, the adjusted CFU was fixed at 1 for time 0. Means and standard deviations of the adjusted CFU are shown. While bacterial load was controlled in SH flies from 6 h onwards, it exponentially rose in DH flies. Of note, sterile wounding did not lead to bacterial infection (data not shown). Statistical analyses have been done using the Log Rank test method (***P < 0.001).

To analyze a possible interaction between trauma and infection in Drosophila, we compared the survival rate of 1 week wild-type adult females infected by pricking with *Pe* (Simple Hit flies, SH) to that of litter-mates that have been wounded in the thorax using a sterile needle before being infected (Double Hit flies, DH). Fly survival was analyzed during the first 50 hours following manipulation. As shown in Fig. 1a, DH flies displayed a much reduced viability compared to SH flies (*P* < 2e^−16^), and the effect was more pronounced during the first 20 hours after trauma and infection. Control sham flies, which were first wounded and then pricked with a sterile needle instead of being infected with *Pe*, displayed no mortality during this time course. Moreover, when a heat-killed bacterium culture was used, DH flies displayed no acute mortality compared to SH ones (see Supplementary Figure S1a). Thus, trauma increased flies mortality during virulent infection and not upon infection with dead bacteria. This indicates that the trauma consecutive to thorax wounding increases the susceptibility of flies to *Pe* infection. In support of this assumption, we observed that this effect was proportional to the size of the wound. Indeed, using a smaller needle for the aseptic wound led to reduced effect on the mortality of flies (Fig. 1b). Importantly, the timing between the trauma and the infectious events had a major impact in the observed susceptibility of DH flies. Indeed, when infection was inflicted more than 1 hour after the sterile injury, DH flies did not display increased mortality to infection compared to SH ones (not shown), suggesting that DH flies susceptibility may be restricted to the acute phase of the inflammatory response. Of note, *Pe* is a highly virulent pathogen for Drosophila melanogaster^13^ and all SH and DH flies died within 5 days after wounding while control sham flies did survive during this time course (see Supplementary Figure S1b).

Trauma induced increased mortality upon infection could be due either to a reduced ability of flies to eliminate the pathogen (reduced resistance) or to a lower tolerance to *Pe*. Reduced resistance should lead to increased bacterial load in DH flies compared to SH ones. We therefore analyzed the bacterial load of SH, DH and control flies during the first 24 hours following infection (Fig. 1c). Remarkably, while DH and SH flies had receive similar amount of bacteria (approximately 1 000 CFU, not shown), we observed a 5 to 10 fold bacterial load increase respectively in DH and SH flies at 6 hours. While bacterial load was controlled in SH flies at 24 hours, it increased exponentially in DH flies (Fig. 1c). A Mann-Whitney analysis showed that the adjusted CFU of DH flies was significantly higher than the one of SH flies (*P* = 0.01). This indicates that wounding-induced trauma lowers fly resistance to infection.

### Identifying molecular signatures of SH and DH phenotypes using RNAseq

**Figure 2:**
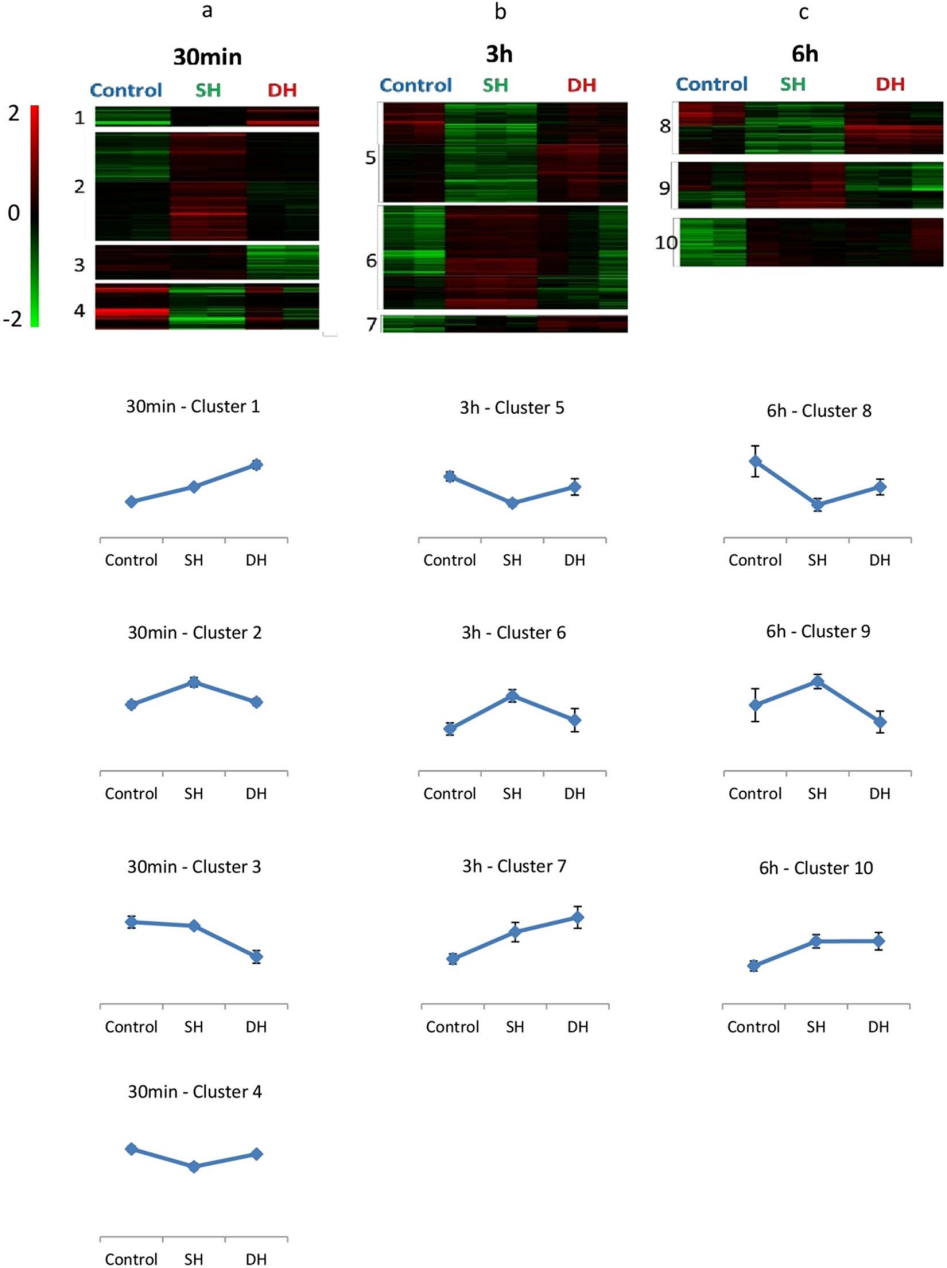
Expression profiling of SH, DH and control flies. Expression profiles of genes whose transcripts levels changed significantly between SH, DH and control at 30 minutes (a), 3 h (b) or 6 h (c). Top: each raw represents a single gene ((**a**) 645 genes, (**b**) 786 genes and (**c**) 450 genes). Columns represent the 3 different conditions: control, SH and DH. Clustering has been done using the method of Kmeans. Normalized log2 expression values were color coded according to the legend at the top. Bottom: expression profiles specific for each cluster. Each curve represents the average expression of the genes within the corresponding cluster. Error bars indicate standard deviations of average expression.

To investigate the mechanisms that may lead to reduced resistance to *Pe* infection in DH flies, we analyzed and compared the transcriptome landscape of SH, DH and control flies. We focused on the early response to trauma and infection challenges and therefore determined gene expression at 30 minutes, 3 hours and 6 hours after treatment. Single end mRNA sequencing was performed on mRNA purified from whole Drosophila wild-type females at the three time points. After sequence read alignment on the reference genome, read counts were normalized to identify differentially expressed genes using both edgeR^14^ and DEseq2^15^ tools. We considered genes identified by both methods in order to increase the stringency of the analysis. Through this comparative analysis of the data, corrected for multi-testing with a FDR of 5%, we found 645, 786 and 449 genes differentially expressed at least in one group (SH, DH or control) at 30 minutes, 3 hours or 6 hours, respectively (see Supplementary Table S1). For each time point, differentially expressed genes were clustered into similar expression profiles using Kmeans clustering, identifying 4 clusters at 30 minutes, 3 clusters at 3 hours and 3 clusters at 6 hours (see Supplementary Table S2 and Fig. 2).

To validate the RNAseq expression data by real time quantitative PCR (RT-qPCR), several genes with diverse expression profiles were selected. In all cases the expression profile observed in the RNAseq were confirmed (see Supplementary Figure S2).

To analyze the functional characteristics of genes with similar expression pattern, we searched for biased representation of gene function annotations within individual expression clusters using Flymine (http://www.flymine.org)^16^ and focused on enrichment for Gene Ontology terms (Table 1). We also used i-*cis*Target method^17^ to identify transcriptional regulatory motifs enriched in these genes clusters, and to propose putative TFs involved in their co-regulation (Table 1). A detailed description of the functional annotations associated with each cluster is given in the supplementary material (see supplementary text T1). We focus here on the main biological processes unraveled.

**Table 1:** Functional annotation enrichments for gene clusters. Gene clusters described in Fig. 2 were analysed for Gene Ontology term enrichment (GO terms) using flymine online database (http://www.flymine.org/). Significant P values (cutoff 0.05) are indicated (see Supplementary Table S3 for details) and the number of genes associated to corresponding GO terms is displayed (Nb genes/GO Term). TF-binding motifs enriched in non-coding regions of gene clusters were identified using i-*cis*Target (https://gbiomed.kuleuven.be/apps/lcb/i-cisTarget/). The predicted corresponding TF and the NES score of the most representative motifs (cut off 5) are indicated (see Supplementary Table S4 for details).

# Cluster	Timepoint	Nb Genes/Cluster	GO Terms (P.val)	Nb Genes/GO Term	TF predicted (NES score)
**1**	**30 min**	**54**	Response to stress [GO:0006950] (2.10e^−3^)	18	Tbp (10.7) Hsf (8.6)
**2**	**30 min**	**360**	Antibacterial humoral response [GO:0019731] (2.79e^−5^)	12	—
**3**	**30 min**	**84**	Eggshell formation [GO:0030703] (1e^−7^)	12	Tbp (7.5)
			Multi-organism process [GO:0051704] (1.9e^−4^)	26	Taf1 (7.5)
					Dl (5.8)
**4**	**30 min**	**147**	Tissue development [GO:0009888] (0.03)	29	Ken (9.7) Blimp-1 (8.8) Hsf (6.2)
**5**	**3 h**	**355**	Response to stress [GO:0006950] (0.02)	52	Atf3 (5.1) Kay (6.4) Jra (6,4)
**6**	**3 h**	**416**	Egg coat formation [GO:0035803] (4.56e^−7^)	9	Pnr (8.6)
			Transmembrane transport [GO:0055085] (2.41e^−6^)	39	GATAe (8.5)
			Immune response [GO:0006955] (0.03)	26	Srp (7.5)
					Grn (7.0)
**7**	**3 h**	**15**	—	—	—
**8**	**6 h**	**160**	Defense response [GO:0006952] (2.27e^−4^)	19	Tbp (6.3)
					Rel (6.3)
**9**	**6 h**	**158**	Egg coat formation [GO:0035803] (4.05e^−13^)	10	—
**10**	**6 h**	**131**	Cell-cell signaling [GO:0007267] (7.82e^−7^)	25	—

#### Innate immune response is rapidly mounted in SH flies but not in DH ones

As early as 30 minutes after manipulations, genes involved in the anti-bacterial humoral response are up-regulated in SH group compared to both DH and control flies (cluster 2, *P* = 2.79e^−5^) (Table 1), including those coding for Diptericin B (*DptB*), Cecropin A1/A2/C (*CecA1*, *CecA2*, *CecC*), Attacin A/B/C (*AttA*, *AttB*, *AttC*). Cluster 2 also comprise genes encoding the Peptidoglycan-Recognition Protein-SA (*PGRP-SA*) and Peptidoglycan-Recognition Protein-SD (*PGRP-SD*), which are respectively involved in the recognition of Gram positive and Gram negative bacteria molecular pattern^18,19^ and the gene coding for the Spätzle-Processing Enzyme (*SPE*), which activates the Toll signaling pathway through the cleavage of its ligand Spätzle (*Spz*) after an infection^20^. *Turandot A/C* genes (*TotA*, *TotC*) are also part of cluster 2. They are known to be strongly induced upon bacterial challenge^21^. This suggests that the protective innate immune response is rapidly mounted in SH flies, but not in DH ones.

The expression of innate immune response genes was maintained at 3 hours at higher levels in SH flies compared to DH ones, similarly to what was observed at 30 minutes (cluster 6, Table 1). These included antimicrobial peptide coding genes such as *Diptericin* (*Dpt*), *Diptericin B* (*DptB*), *Drosocin* (*Dro*), *CecA1*, *CecA2*, *CecC*, *Defensin* (*Def*) and *Metchnikowin* (*Mtk*), as well as the known regulators of the immune response *PGRP-SC1a*, *PGRP-SC1b*, *PGRP-SC2*. The i-*cis*Target analysis pointed out GATA type TF binding motifs in non-coding region of cluster 6 genes, suggesting the involvement of this TF family in the regulation of immune response as previously reported^22^.

#### Up regulation of stress response related genes in DH flies

Cluster 1 contained genes highly expressed in DH flies compared to both SH and control ones (Fig. 2a). This gene set was enriched in GO annotation associated with ‘response to stress’ (*P* = 2.10e^−3^) (Table 1). Of note, among these genes, several are known actors of the JNK signaling pathway (*Gadd45*, *Hsromega* and *Cabut*) while a number of genes participating in the heat shock response were also retrieved (*Hsp70Ab*, *Hsp70Bb*, *Hsp70Aa*, *Hsp70Ba*, *Hsp70Bc*, *Hsp70Bbb*). Interestingly, among the position weight matrices (PWM) significantly outlined by i-*cis*Target within cluster 1, one was predicted to bind the Heat Shock Factor (Hsf, Table 1). Overall, these functional annotations suggest that combining trauma and infection in DH flies exacerbates the stress response compared to the other situations, where trauma and infection were inflicted separately to the flies.

At 3 hours the term ‘response to stress’ was found enriched in cluster 5 (*P* = 0.02, Table 1) which comprises genes up regulated in both DH and control flies compared to SH ones. As for cluster 1, several genes of this set belong to the JNK pathway: *Gadd45*, *Jun-related antigen* (*Jra/dJun*, the drosophila Jun orthologous gene), *Cabut*, *Puckered* (*Puc*) and *Hsromega*. Noteworthy, binding motifs for the bZip TFs ATF3 and Kayak (Kay/dFos, the drosophila Fos homologue), which are known heterodimers of Jra/dJun as well as for Jra/dJun itself, are identified in the non-coding region of this set of genes by the i-*cis*Target method. This suggests that 3 hours after trauma and infection, DH flies maintain an exacerbated stress response compared to SH ones -most probably mediated by JNK pathway- and that control flies, which were subjected to trauma alone, have also mounted a stress response, although delayed compared to DH ones.

#### CrebA targets are induced in SH flies

Importantly, a significant proportion of genes previously identified as potential transcriptional targets of the CrebA/Creb3-like protein, a known regulator of cells secretory capacity^23^, were up regulated in SH flies at 3 hours (cluster 6) (hypergeometric test; *P* = 4.90e^−13^) (see Supplementary Table S5). As a matter of facts, genes annotated for transmembrane transport are enriched in this cluster (*P* = 2.41e^−6^). In addition, although the gene coding for the CrebA TF was not included in the cluster 6, relaxing the stringency of the differential expression analysis by considering genes identified only by edgeR, pointed out the *CrebA* gene as down-regulated in DH flies compared to SH ones (see Supplementary Table S2). RT-qPCR confirmed the expression profile of *CrebA* and several of its targets (*CG5885*, *Sec*. 6*1beta*, *Spase25* and *TRAM*, see Supplementary Figure S2b). This suggests that infection alone triggers a *CrebA* mediated up-regulation of secretory pathway and that this process may not be activated upon trauma.

Overall, this data mining suggests that flies subjected to both trauma and infection display an immediate activation of stress response genes, which is exacerbated compared to that of flies infected only. This analysis also underlines the possible involvement of the JNK pathway in this process. In addition the innate immune response appeared to be rapidly mounted in SH flies which was not the case in DH ones. This could explain their reduced resistance to infection. Interestingly, in SH flies, genes related to transmembrane transport were actively transcribed and this process might depend on the CrebA TF. Importantly the up regulation of *Jra/dJun* and some of its targets in DH flies and of *CrebA* targets in the SH group was indeed validated by RT-qPCR (see Supplementary Figure S2).

### Functional analysis of Jra/dJun and CrebA TFs

To analyze the functional involvement of the JNK and CrebA pathways in the differential susceptibility of DH and SH flies to *Pe* infection, we inhibited and/or overexpressed the TFs Jra/dJun (the downstream target of JNK pathway) and CrebA, which have been found differentially expressed in DH and SH flies.

The implication of these TFs in the reduced survival of DH flies was tested by genetically manipulating their activity in whole flies using the GeneSwitch system^24^. Briefly, this system allows the expression of a transgene to be induced by RU486 feeding and therefore permits temporal control of gene expression. We used the Tubulin > GeneSwitch driver in order to drive expression of transgenes in whole individuals. Induction at adulthood allowed us to bypass potential secondary effects that may be caused by inhibiting or activating the pathways during embryonic development, larval life or metamorphosis. In addition, the GeneSwitch system enables comparing individuals of the same genetic background. We verified that, whatever its concentration (from 20 µg to 200 µg), RU486 alone, without inducing a transgene expression, has no effect on the survival rates of DH, SH and control flies (see Supplementary Figure S3).

**Figure 3:**
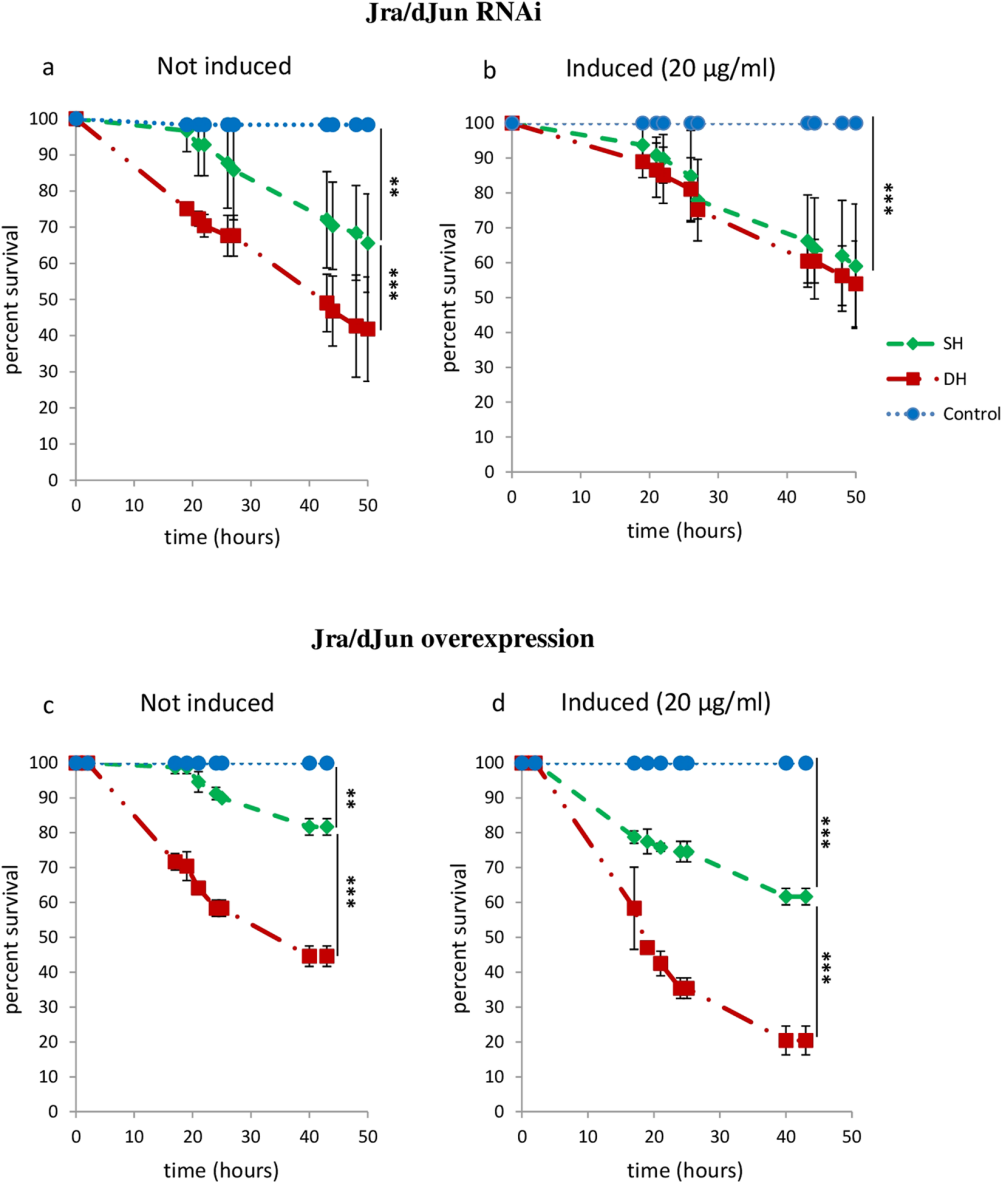
Impact of Jra/dJun modulation of expression on survival of SH and DH flies. Sterile wounding and/or *Pe* infection were performed on 1 week old adult females of Tubulin > GeneSwith/+; UAS-*dJun* ^RNAi^/+ (*Jra/dJun* RNAi, (**a**,**b**) and Tubulin > GeneSwith/+; UAS-*Jra*/+ (*Jra/dJun* overexpression) genotypes in the absence (**a**,**c**: not induced) or in the presence (**b**,**d**: induced) of RU486 at indicated concentrations. Fly survival was surveyed up to 50 hours after treatments. (**a**,**b**) *Jra/dJun* inhibition improved the survival of DH flies but did not impact that of SH ones (see also Supplementary Figure S3). (**c**,**d**) *Jra/dJun* overexpression was deleterious for both DH and SH flies (see also Supplementary Figure S3). Each group consisted of 120 flies observed among 3 different experiments (40 flies/group/experiment). Statistical analyses were performed using the Log Rank test method (**P < 0.01; ***P < 0.001). Means of survival are shown, and error bars represent standard deviations.

We first measured the survival rate of flies subjected to RNA interference mediated inactivation of *Jra/dJun* upon SH and DH challenge as well as in control sham flies. RT-qPCR confirmed the efficiency of *Jra/dJun* inhibition upon RU486 induction (supplementary figure S4). *Jra/dJun* inhibition had no significant effect on survival of control and SH flies, while it almost completely rescued DH flies survival whose rate reached that observed for the SH ones (Fig. 3b and Supplementary Figure S4). This supports *Jra/dJun* involvement in the higher sensibility to *Pe* infection of DH flies compared to SH ones. This was confirmed by the effect of *Jra/dJun* overexpression, which worsened both SH and DH flies survival (Fig. 3d and Supplementary Figure S4). Thus, in wounded and infected DH flies, the activation of the JNK pathway was detrimental and appeared responsible, at least in part, for their reduced resistance to *Pe* infection.

**Figure 4:**
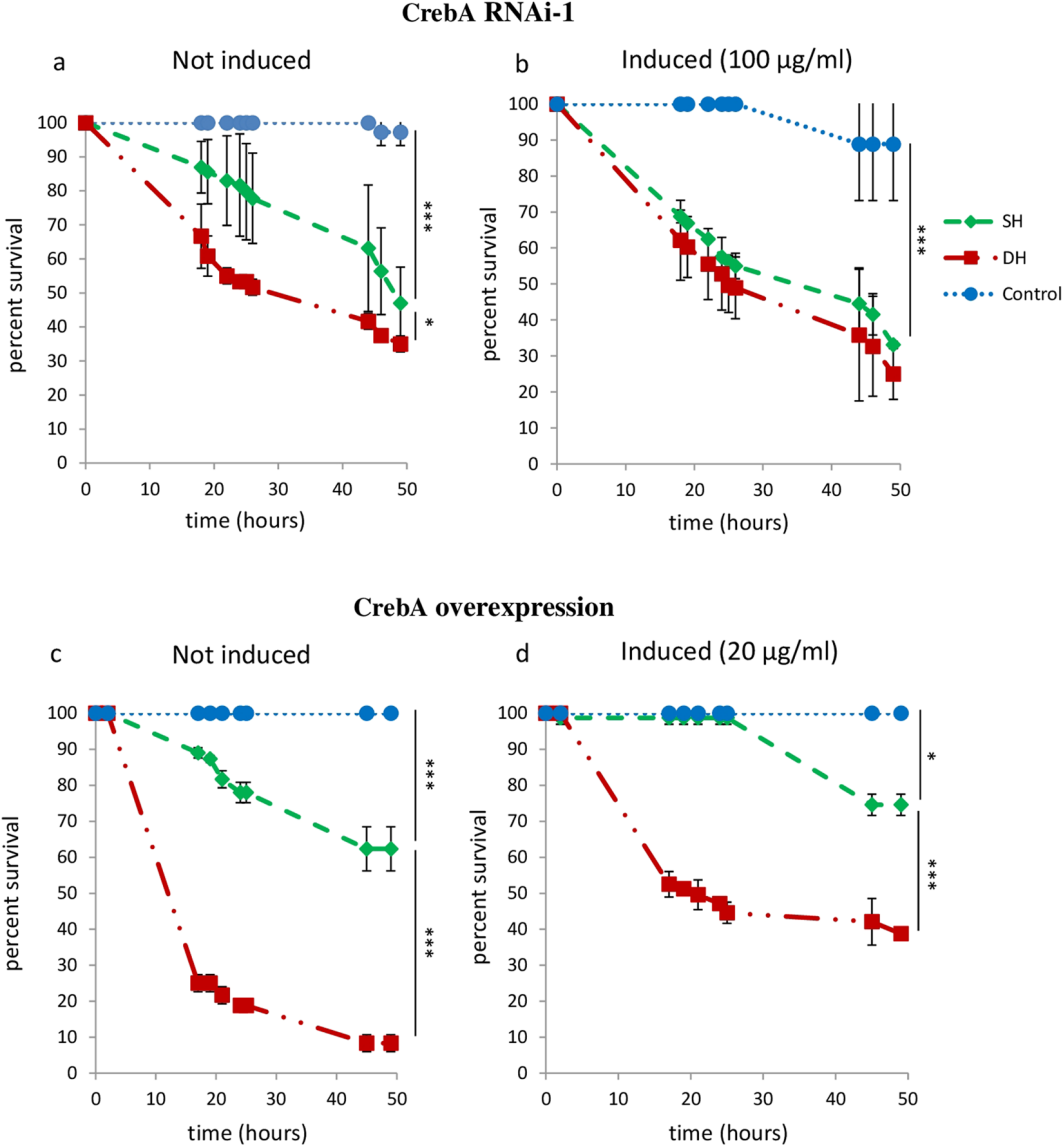
Impact of the modulation of CrebA expression on survival of SH and DH flies. Sterile wounding and/or *Pe* infection were performed on 1 week old adult females of Tubulin > GeneSwith/+; UAS-*CrebA* ^RNAi^/+ (*CrebA* RNAi-1; (**a**,**b**) and Tubulin > GeneSwith /+; UAS-*CrebA*/+ (CrebA overexpression; (**b**,**c**) genotypes in the absence (**a**,**c**: not induced) or in the presence (**b**,**d**: induced) of RU486 at indicated concentrations. Fly survival was observed up to 50 hours after treatment. (**a**,**b**) *CrebA* inhibition worsened the survival of SH flies but did not impact that of DH ones (see Supplementary Figure S4). (**c**,**d**) *CrebA* overexpression improved both DH and SH flies survival (see Supplementary Figure S4). Each group consisted of 120 flies observed among 3 different experiments (40 flies/group/experiment). Statistical analyses were performed using the Log Rank test method (**P < 0.01; ***P < 0.001). Means of survival are shown, and error bars represent standard deviations.

We next tested the impact of modulating *CrebA* gene function using the same GeneSwitch system (Fig. 4). While the decrease of CrebA activity had no effect on the survival rate of DH flies (Fig. 4b and Supplementary Figure S5), it had a major impact on the survival of SH ones, which behaved like DH flies. This was observed using two independent RNAi lines (Supplementary Figure S5), excluding the possibility of off targets effects. This indicates that *CrebA* has a major function in *Pe* infected fly survival and suggests that reduced DH fly resistance to infection may be at least in part due to reduced *CrebA* function. The effect observed by inducing *CrebA* overexpression in whole flies supports this view. Indeed, the gain of function of this TF improved the survival rate of both DH and SH flies (Fig. 4d and Supplementary Figure S5) supporting the idea that *CrebA* plays a main role to fight *Pe* infection.

### *CrebA* acts downstream of *Jra/dJun*

**Figure 5:**
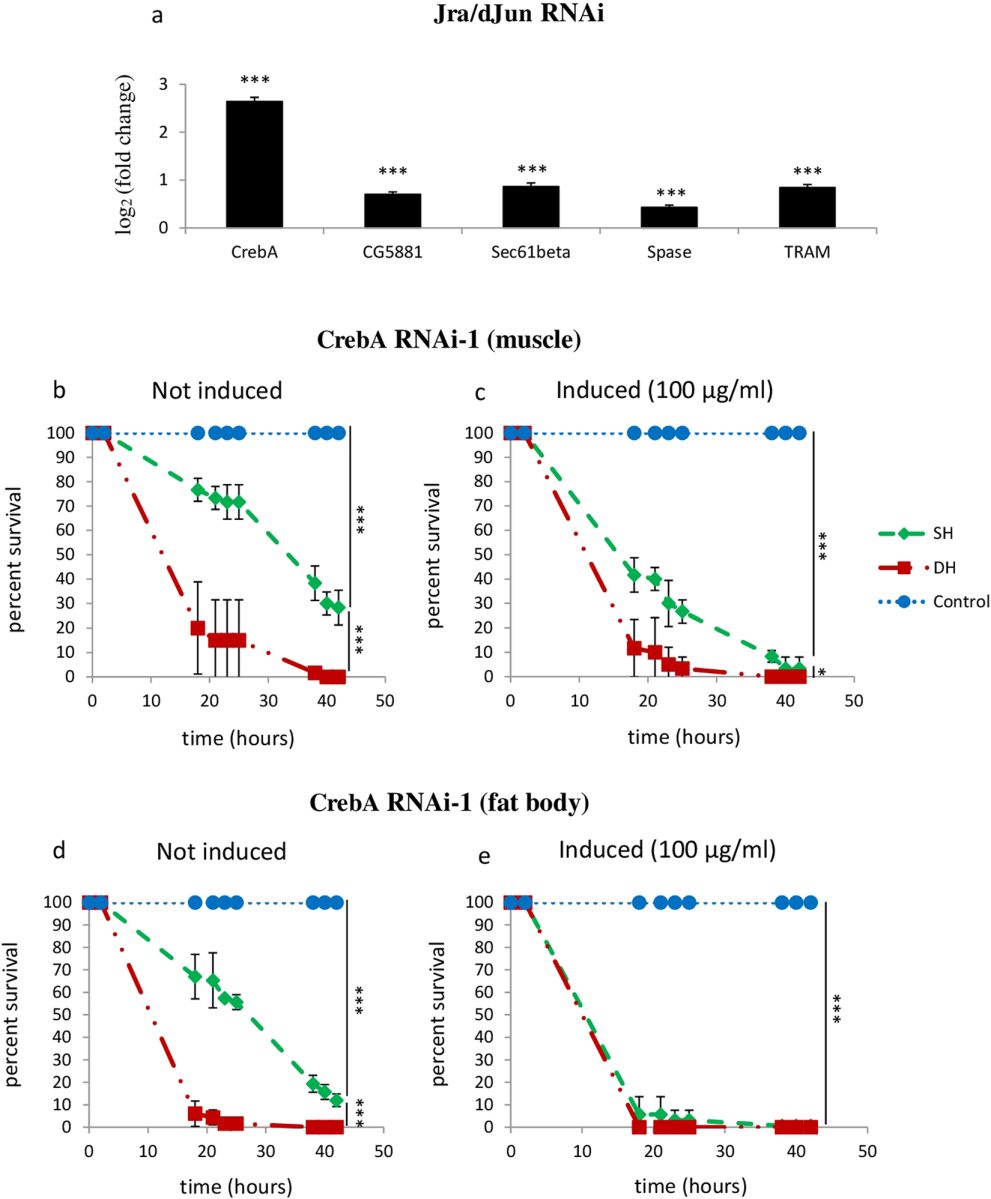
*CrebA* acts downstream of Jra/dJun and its function is required in both muscles and fat body. (**a**) *CrebA* acts downstream of *Jra/djun*. *CrebA* and CrebA targets expression levels was measured by RT-qPCR on Tubulin > GeneSwith/+; UAS-*dJun* ^RNAi^/+ individuals induced or not using RU486 at 20 μg/ml. Black bars represent mean value of log2 (fold change) observed in Jra/dJun RNAi mediated knockdown (induced condition) normalized to non-induced condition. P value was calculated using the T-Test method (***P < 0.001; CrebA: *P* = 2.29e^−5^; CG5881: *P* = 6.63e^−5^; Sec. 61beta: *P* = 1.79e^−4^; Spase: *P* = 4.89e^−4^; TRAM: *P* = 2.03e^−4^). (**b**–**e**) Impact of muscle-specific or fat body specific loss of function of *CrebA* expression on survival of SH and DH flies. Sterile wounding and/or *Pe* infection were performed on 1 week old adult females of MHC > GeneSwith/+; UAS-*CrebA* ^RNAi^/+ (*CrebA* RNAi-1 (muscle)) (**b**,**c**) and Lsp2 > GeneSwith/+; UAS-*CrebA* ^RNAi^/+ (*CrebA* RNAi-1 (fat body)) (**d**,**e**) genotypes in the absence (**b**,**d**: not induced) or in the presence (c, e: induced) of RU486 at indicated concentrations. Fly survival was observed up to 50 hours after treatment. (**b**,**c**) Muscle-specific *CrebA* inhibition worsened the survival of SH flies but did not impact that of DH ones. (**d**,**e**): Fat body-specific *CrebA* inhibition worsened SH flies survival. Each group consisted of 120 flies observed among 2 different experiments (60 flies/group/experiment). Statistical analyses were performed using the Log Rank test method (*P < 0.05; ***P < 0.001). Means of survival are shown, and error bars represent standard deviations.

We next wondered if there is a link between JNK and CrebA activity. One plausible scenario was that activation of the JNK pathway in DH flies lead to a reduced *CrebA* expression, which in turn impacts on organismal resistance to infection^25^. We therefore analyzed the expression of *CrebA* and some of its putative targets following *Jra/dJun* knockdown. As shown in Fig. 5a, *Jra/djun* inhibition by RNA interference is accompanied by an important increase in the transcripts levels of both *CrebA* and its targets, indicating that *Jra/dJun* behaves as a transcriptional repressor of *CrebA*.

### *CrebA* function is required in both muscles and fat body

The reduced resistance of SH flies to *Pe* infection following ubiquitous loss of *CrebA* function and the reverse effect observed upon *CrebA* gain of function unraveled a novel function of *CrebA* in innate immunity. In order to further characterize *CrebA* activity in this process we aimed at unraveling the tissue(s) in which it is required. Muscles and fat body represented the most likely candidates and were tested with respective GeneSwitch drivers. As shown in Fig. 5c–e knocking down *CrebA* in either of these tissues impaired SH flies survival, indicating that the function of this TF is required in both tissues for accurate *Pe* resistance.

## Discussion

We have shown that thorax wounding induces a reduced resistance to *Pseudomonas entomophila* challenge and is accompanied by increased host mortality upon infection. Our results thus confirm the observation made by Chambers *et al*. (2015)^26^. Moreover, we demonstrated that this susceptibility is proportional to the size of the trauma performed. Importantly, survival rates differences between DH (wounded and infected) and SH (infected) flies were observed in the earlier phase of the experiments (up to 20 hours after trauma and infection).

We investigated the underlying mechanisms of DH flies susceptibility to infection by comparing the transcriptome landscape of DH, SH, and control flies during the early steps of the process. We observed that the anti-bacterial immune response was rapidly mounted in SH flies but not in DH ones. Conversely, genes with increased expression in DH flies compared to SH ones were significantly enriched for stress related annotations, including heat shock related genes and members of the JNK pathway. Therefore, our results point out an interaction between trauma and infection in flies, which culminated in a reduced resistance to infection following trauma and may be mediated by stress pathway related genes.

We further demonstrated that the JNK pathway plays a central role in the DH phenotype. Indeed, members of the pathway were actively transcribed in DH flies, as soon as 30 minutes after treatment, and reduction of *Jra/dJun* activity rescued the increased mortality of DH flies, while its increase worsened the survival of SH ones. This indicates that JNK pathway activation, which was stronger and appeared earlier in DH flies, is instrumental in their reduced resistance to an infection following trauma. Of note, the JNK pathway is involved in both inflammation and infection in numerous model organisms, from flies to mammals. For instance, in flies, signaling through the JNK pathway has been implicated in the transcriptional response to various microbial agents^27^ and is activated by ROS and required for regeneration that maintains tissue homeostasis following tissue damage^28^. In human monocytes, JNK pathway activation leads to the production of inflammatory cytokines^29^, whereas it induces cell oxidative damage in human endothelial cells^30^. In DH flies, the JNK pathway may be activated independently both by sterile injury and by bacterial infection then leading to its hyper-activation that could have deleterious effects including inappropriate response to infection.

We also provided evidence that *CrebA* is involved in the decreased survival of DH flies. First, the set of overexpressed genes in SH flies at 3 hours was enriched for the CrebA TF targets, suggesting that DH condition prevents their activation. Second, we demonstrated that the decrease of *CrebA* activity reduced the survival of SH flies, which became similar to that of DH flies, whereas *CrebA* overexpression improved the survival rate of both DH and SH flies. This demonstrates that decreased *CrebA* activity in DH is central to their higher sensitivity to *Pe* infection. Interestingly, in mammals, the CrebA/CrebH TF is known to be involved in systemic inflammatory response^31^. In addition, multiple members of the CrebA family have been implicated in signaling pathways downstream inflammation and infection^32^, suggesting a conserved function for CrebA in the inflammatory response.

Since manipulating *CrebA* function affects the survival of SH flies - in the absence of septic injury- our results unravel its function in innate immunity in flies which, to the best of our knowledge, was not characterized so far. Further supporting this newly discovered function, a recent report identified an SNP in *CrebA* associated to decreased resistance to *Pseudomonas Aeruginosa* infection in the Drosophila Genetic Reference Panel^33^. This may prompt further investigations to understand the molecular basis of *CrebA* implication in the fly innate immune system. Of note, CrebA was shown to regulate genes of the secretory pathway^23^ and potential CrebA targets were identified in this study suggesting that CrebA is involved in innate immunity through cell secretion process.

We also provide evidences that the JNK pathway activation is instrumental in the repression of *CrebA* thus establishing a link between the effect of the JNK pathway and that of *CrebA* on DH flies survival. The activation of the stress response pathway thus appears to lower *CrebA* expression, what might impinge on the ability of flies to fight *Pseudomonas entomophila* infection.

Finally we establish that *CrebA* function in SH flies is required in both muscles and fat body, two tissues central for innate immunity in flies^12^^,^^34,35^. It should be stressed that in DH flies the sterile wounding was inflicted in the thorax and may therefore mainly affect the indirect flight muscles (IFMs). As a matter of facts, muscles were demonstrated to provide protection during microbial infection^34^. Furthermore, it has been shown previously that structural components of muscles are involved in resistance to thoracic septic wounding^12^. This suggests that muscles *per se* have a central role in the protection against inflammation and infection. Indeed, a recent study demonstrated that thorax injury, but not abdominal one, lowers resistance to infections in flies^26^, confirming that muscles may have a specific function in the organismal response to sterile inflammation. Our results go a step further by identifying *CrebA* requirement in this tissue and by suggesting that it may be dowregulated via the JNK pathway upon traumatic injury.

To summarize, our results establish that in flies, like in vertebrates, combined trauma and infection is deleterious for survival. In addition we demonstrated a new role for *CrebA* in innate immunity in flies, established that the JNK pathway and *CrebA* are central in the reduced survival of the DH flies and provide evidence that *CrebA* is down-regulated by the JNK pathway following traumatic injury. Since the signaling pathways unraveled here are conserved between Drosophila and mammals, our results may prompt to investigate their involvement in either the hyper-inflammatory response or the immunosuppression leading to morbidity and mortality in sepsis patients.

## Material and Methods

### Fly strains and maintenance

Fly rearing and crosses were performed on standard cornmeal agar yeast food and the flies were raised at 25 °C. The *w* ^*1118*^; canton S isogenic line (*w* ^*1118*^)^36^ was used as a wild-type genetic background. In the study of survival of transgenic lines, the *w* ^*1118*^ strain was systematically considered as positive control of the DH experiment; the reproducibility of *w* ^*1118*^ flies survival rates in DH conditions allowed to ensure a constant pathogenicity of the *Pseudomonas entomophila* bacteria between experiments. Infections were carried out at a similar time of day across experimental replicates in order to avoid the influence of circadian rhythm on the drosophila immunity response.

The following transgenic flies were obtained as indicated for use in this study: UAS-*Jra* ^RNAi^ (VDRC# 107997); UAS- *CrebA* ^RNAi^ (TRiP HMJ02218, Bloomington # 42562 (*CrebA* RNAi-1); TRiP JF02189, Bloomington # 31900 (*CrebA* RNAi-2)); UAS-CrebA (gift from D. Andrew); UAS-Jra (Bloomington# 7216). We used the ubiquitous tubulin-GeneSwitch Gal4 (Tub-GS, Flybase # Fbti0145090) and the muscle specific MHC-GeneSwitch Gal4 (MHC-GS)^24^. For fat body restricted expression, we generated a GeneSwitch driver (Lsp2-GeneSwitch Gal 4) by inserting a 3,1 kb promoter fragment from the Lsp2 gene^37^ in the pAttB-Sfi-GALGS vector and recovered insertions at attp40. The different Upstream Activating Sequence (UAS) transgenes were activated by adding RU486 (Mifepristone, SIGMA) in the food at 20 μg/ml, 50 μg/ml, 100 μg/ml or 200 μg/ml in the food at adulthood^36^.

### Bacterial culture

The Gram-negative bacteria *Pseudomonas entomophila* was a kind gift of Dr A. Gallet. *Pe* suspension was prepared in Luria-Bertani broth from frozen glycerol stock. Bacteria were spread on LB agar plate supplemented by 1% of dry milk and were grown overnight at 30 °C. The presence of a translucent halo around a colony allows to easily spot a strong caseinase activity of the bacterial colony which is a mark of their virulence. Starter cultures were made up by inoculating 40 ml of LB into a sterile 250 ml conical flask with a single colony from the agar LB plate and were grown up 8 h in an orbital shaker set at 250 revolutions per minute (rpm) at 30 °C. Overnight liquid cultures were prepared by inoculating 187.5 ml of LB broth in a sterile 1 l conical flask with 12.5 ml of starter culture and were grown at 250 rpm at 30 °C. Bacteria were allowed to grow up to an optical density at 600 nm (OD_600_) comprised between 7 and 10 and the OD_600_ was then adjusted to 1 for infections.

### Sterile wounding and infection

All experiments used 7 to 10 days-old adult females flies manipulated at room temperature (23 °C) upon CO_2_ anesthesia. Flies were pricked with tungsten needles on thorax side. The sterile injuries (clean injuries, CI) were performed by pricking flies with a 0.3 mm diameter needle (large needle). A 0.1 mm diameter needle (small needle) dipped into the 1 OD_600_ bacteria culture was used for the infection. Double hit (DH) flies were pricked with sterile needle and infectious needle on each side of the thorax. The control sham flies were pricked with both sterile large and small needles^37^.

### Fly survival and bacterial charge measurement

Fly survival was determined on groups of 40 manipulated flies (in 4 batches of 10 flies per vial) for up to 50 h at 25 °C. Flies that died within 2 h after manipulation were excluded from the analysis. To determine the bacterial load in manipulated flies, a sample of 3 flies per group were collected at 0 h, 6 h and 24 h after manipulation and were crushed in 1 ml of LB broth. 10-fold serial dilutions of samples suspension were made and 100 µl plated on LB agar. Colonies were counted after 24 h at 30 °C.

### Statistical analysis and fly survival

Statistical analysis of survival rates between different batches of manipulated flies was performed using R project software (http://www.r-project.org/). For comparing fly groups, the survival curves were analyzed by the Log Rank test method^39^ in order to test the null hypothesis that the survival kinetics of the different populations of flies (the probability of the “death” event at any time point between the different batches) are equivalent. In each group and for each time point, the observed number of death and the expected number of death predicted by the null hypothesis were calculated. Then, total numbers of expected deaths (E) and observed deaths (O) in each group were calculated. The method next relies on the use of a χ^2^ test of the null hypothesis, based on the calculation of the sum of (O-E)^2^/E for each group, the degrees of freedom being the number of groups minus one. The P-value was calculated for the overall time course, from a Table of the χ^2^ distribution. This P-value represents the probability of flies randomly selected from those predicted by the null hypothesis to have survival curves different to those actually observed. Difference between the groups was considered statistically significant if the P-value was lower than 0.01.

### RNA sequencing

Total RNA was isolated, using TriZOL chloroform extraction (Ambion), from 15 flies for each condition (i.e. sham, SH and DH) divided into 3 biological replicates at 30 min, 3 h and 6 h post experiment. mRNA population was purified through a polyA selection and was fragmented into sequences of 200 nucleotides. After cDNA synthesis and its amplification by PCR, libraries were normalized and sequenced using Illumina NextSeq-500 sequencer, through a single-end strategy (http://www.illumina.com/systems/nextseqsequencer.html).

### RNA-seq analysis

Illumina sequence files were converted into FASTQ format. The short sequence reads (75 nucleotides) were aligned to the Drosophila reference genome (*dm3*; http://genome.ucsc.edu/) and assigned to genes using Bowtie (version 2)^40^ with the following parameters:

–end-to-end–sensitive–no-unal -t -k 1 -q -U. Preset ‘–sensitive’ option ensures good sensitivity and fast processing. End-to-end argument is required in order to align the entire read length. No-unal argument allows discarding reads that failed to align and -k 1 argument allows read to align once.

We used the Subread package (R statistical tool; http://www.r-project.org/) to count aligned reads. For each time point (30 min, 3 h and 6 h), differentially expressed genes were identified by the use of the R packages edgeR^14^ and DESeq2^15^. We used no fold change filtering and results were corrected for multi-testing by the method of the False Discovery Rate at the 5% level. Clustering were done using TIGR Multiexperiment Viewer tool (MeV)^41^. Differentially expressed genes were clustered using the unsupervised classification method of the Kmeans^42^. Functional annotation enrichment for Gene Ontology (GO) terms were realized using Flymine database [http://www.flymine.org/]^16^. Benjamini-Hochberg corrected P values of less than 0.05 were considered significant. We also used i-*cis*Target tool^17^ to look for enrichment in TF (TF) position weight matrices and potential binding sites in the regulatory regions of co-expressed genes. i-*cis*TargetX computes statistical over representation of DNA motifs and ChIP-seq peaks in the non-coding DNA around sets of genes^17^. The enrichment was considered significant when the Normalized Enrichment Score (NES) was higher than 5.

### Validation of RNA-seq data using RT-qPCR

To confirm RNA-seq results, we have selected 4 differentially expressed genes (DEGs) known to be involved in the JNK pathway (*Hsromega* ^43^, *Cabut* ^44^, *Puckered* ^45^ *and Jra/dJjun* ^46^) and 4 DEGs known to be targets of the CrebA transcription factor (*TRAM*, *CG5885*, *Spase25* and *Sec*. *61beta*)^23^. RT-qPCR was performed to confirm the gene expression changes of these 8 genes in our 3 experimental conditions (Control, SH and DH). Since, when relaxing the stringency of the analysis to edgeR results, *CrebA* gene was retrieved as differentially expressed, RT-qPCR was also performed on this gene. RNA extraction from flies was performed with TRIzol™ Reagent according to the manufacturer’s instructions. Then total RNA was reversely transcribed with the Invitrogen™ SuperScript™ IV VILO™ Master Mix according to the manufacturer’s instructions. The resulting cDNA was then used as a template for qPCR. Gene-specific primers were designed using Primer 3 software (see Supplementary Table S6), while the *rp49* gene was used as the internal control. Reactions were performed using the Applied Biosystems™ *QuantStudio*™ *6* Flex Real-Time PCR System with Power SYBR® Green PCR Master Mix. The qPCR program began at 95 °C for 10 min, followed by 40 cycles of 95 °C for 15 s and 60 °C for 1 min. Fluorescent signals were collected at each polymerization step. Melting curve analysis of amplification products was performed at the end to confirm that a single PCR product was detected. Samples were assayed in triplicate and PCR reactions without the template were performed as negative controls. The comparative Ct method for relative quantification was used to analyze the data^47^.

### Data availability

Microarray data are available in the ArrayExpress database (www.ebi.ac.uk/arrayexpress) under accession number E-MTAB-5516.

## Electronic supplementary material

Supplementary Text and Figures

Supplementary Table S1

Supplementary Table S2

Supplementary Table S3

Supplementary Table S4

Supplementary Table S5

Supplementary Table S6
