# Supplementary material for: Interplay between trauma and Pseudomonas entomophila infection in flies: a central role of the JNK pathway and of CrebA
Source: Sci Rep. 2017 Nov 24;7:16222. doi: 10.1038/s41598-017-14969-7 (PMC5701226; doi:10.1038/s41598-017-14969-7)
Supplement: Supplementary file 1 — Supplementary Text and Figures [file 41598_2017_14969_MOESM1_ESM.pdf]

Interplay between trauma and *Pseudomonas entomophila* infection in flies: a central role of the JNK pathway and of CrebA

Ramy Ragheb[1,2], Alexandre Chuyen[1,2], Magali Torres[1,2], Arnaud Defaye[1,2], Denis Seyres[1,2], Laurent Kremmer[1,2], Nicolas Fernandez-Nunez[1,2], Hervé Tricoire[3], Pascal Rihet[1,2], Catherine Nguyen[1,2], Laurence Röder[1,2] *, Laurent Perrin[1,2,4] *

[1] Inserm UMR_S 1090, TAGC, parc scientifique de Luminy, case 908, 13288 Marseille cedex 9, France.

[2] Aix-Marseille université, TAGC, parc scientifique de Luminy, 13288 Marseille, France.

[3] Université Paris Diderot-Paris 7 /CNRS, UMR8251, 4, rue Marie Andree Lagroua Weill Halle, 75013 Paris cedex 13, France

[4] CNRS, Marseille, France.

*Corresponding authors

a

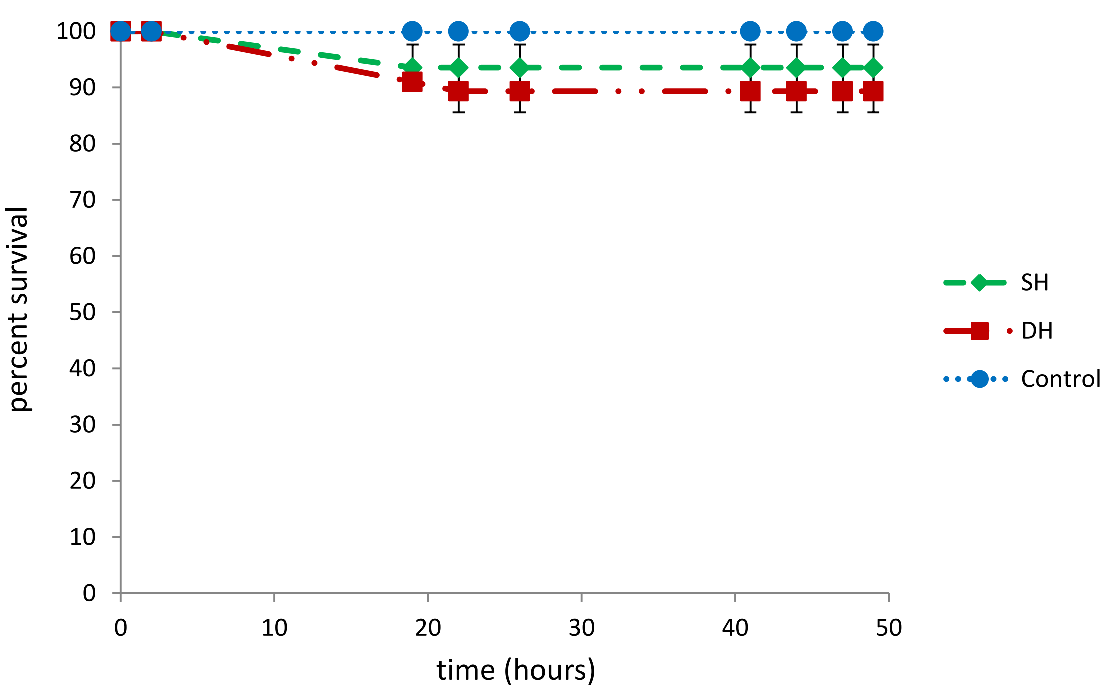

100
90
80
70
60
50
40
30
20
10
0
percent survival
0
10
20
30
40
50
time (hours)
SH
DH
Control

b

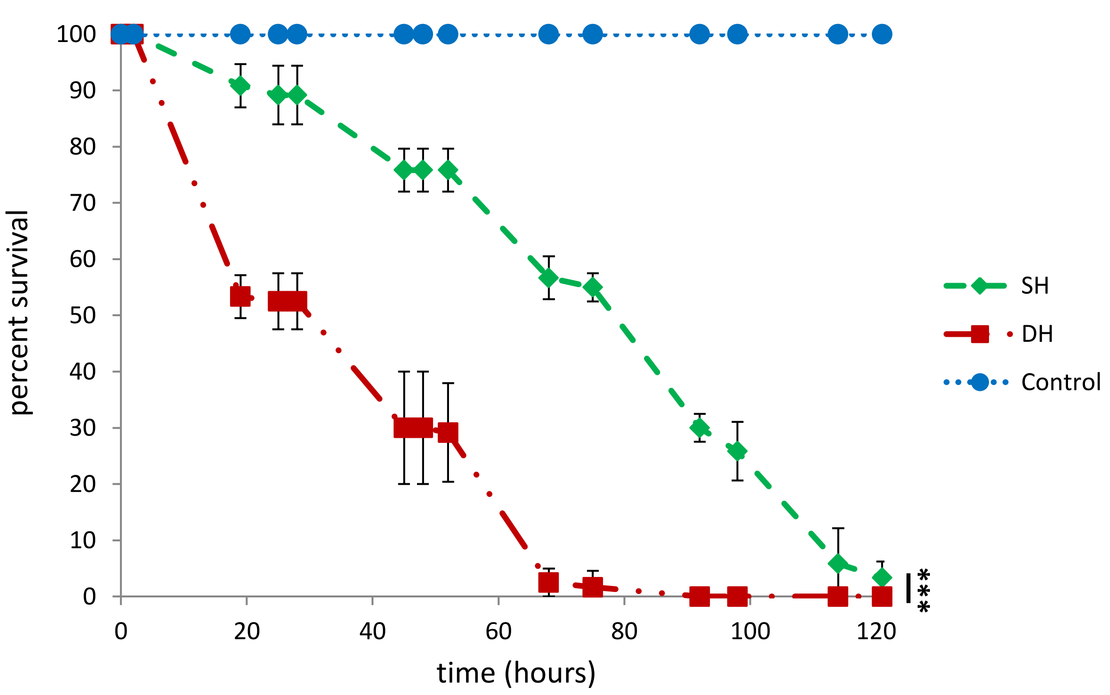

100
90
80
70
60
50
40
30
20
10
0
percent survival
0
20
40
60
80
100
120
time (hours)
***
SH
DH
Control

Supplementary Figure S1: a) **Absence of impact of trauma on the survival of flies pricked with inactivated *Pe*.** Survival rates of control sham (blue), SH (green) and DH (red) flies are shown. Bacteria were heated for 30 min at 95°C prior to infection. Dead bacteria did not significantly affect flies survival rates, and sterile wounding did not compromise their survival rates either. b) **5 days survival observation.** Survival rates of control sham (blue), SH (green) and DH (red) flies was observed during 120 hours (5 days). While *Pe* infection kills all flies within 5 days, DH treatment significantly accelerates lethality. Mean and standard deviations of survival based on 3 experiments each performed on 40 flies per condition is shown. Statistical analyses have been done using the Log Rank test method (*** $P < 0.001$).

a

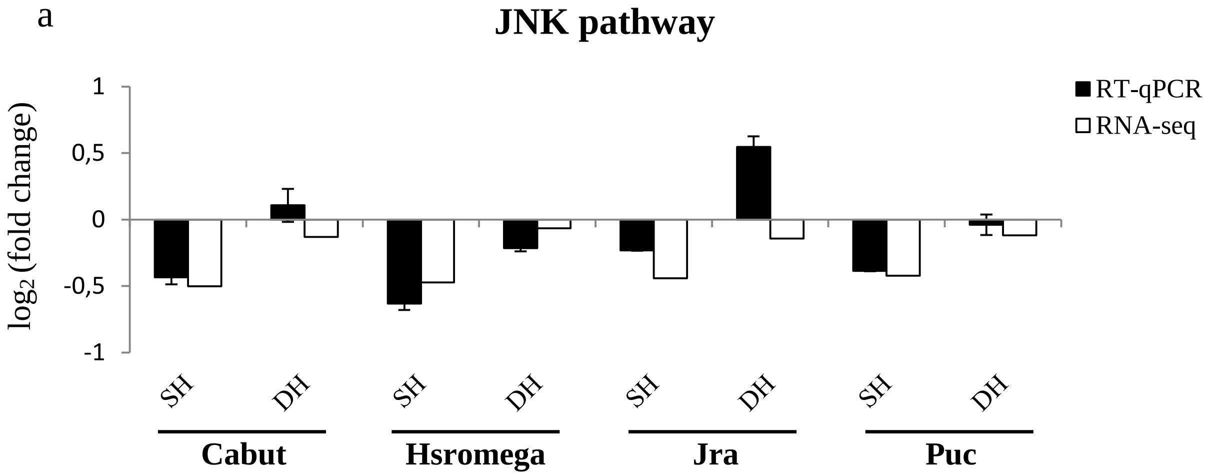
JNK pathway
RT-qPCR
RNA-seq
log2 (fold change)
1
0,5
0
-0,5
-1
SH
DH
Cabut
Hsromega
Jra
Puc

b

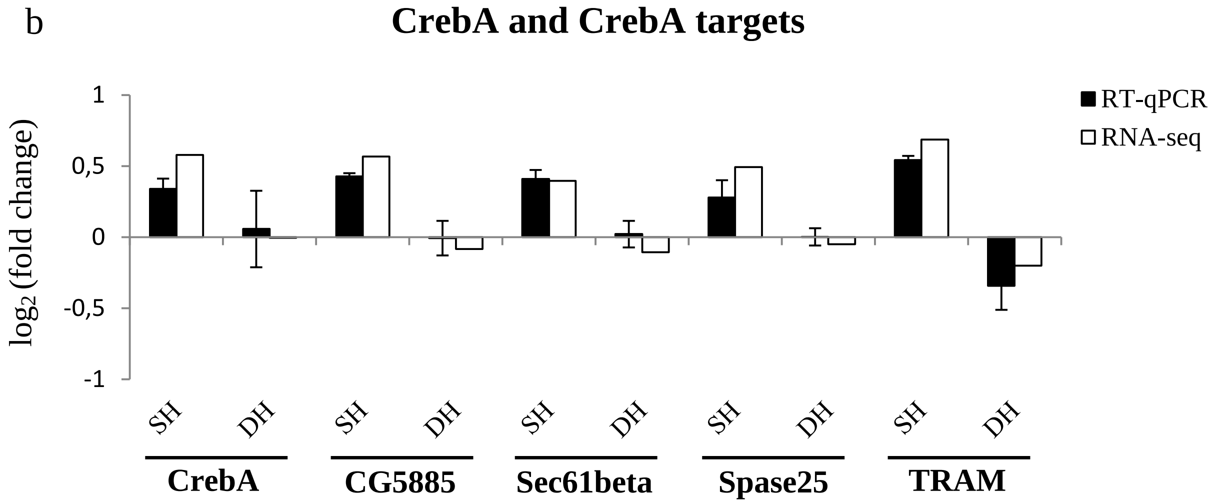
CrebA and CrebA targets
RT-qPCR
RNA-seq
log2 (fold change)
1
0,5
0
-0,5
-1
SH
DH
CrebA
CG5885
Sec61beta
Spase25
TRAM

Supplementary Figure S2: **Validation of RNA-seq data using RT-qPCR.** (a, b) Black bars represent mean values of log2 (fold change), while the white bars represent RNA-seq data observed for SH or DH samples vs controls. Expression level of SH and DH groups were normalized to the control expression level. Three biological replicates were performed for RT-qPCR. RT-qPCR data were analyzed through the comparative Ct method for relative quantification. Error bars represent standard deviation. (a) Bar plots representing log2 (fold change) for 4 JNK pathway-related gene expression. (b) Bar plots representing log2 (fold change) of the expression of genes encoding the CrebA transcription factor and 4 of its known target genes.

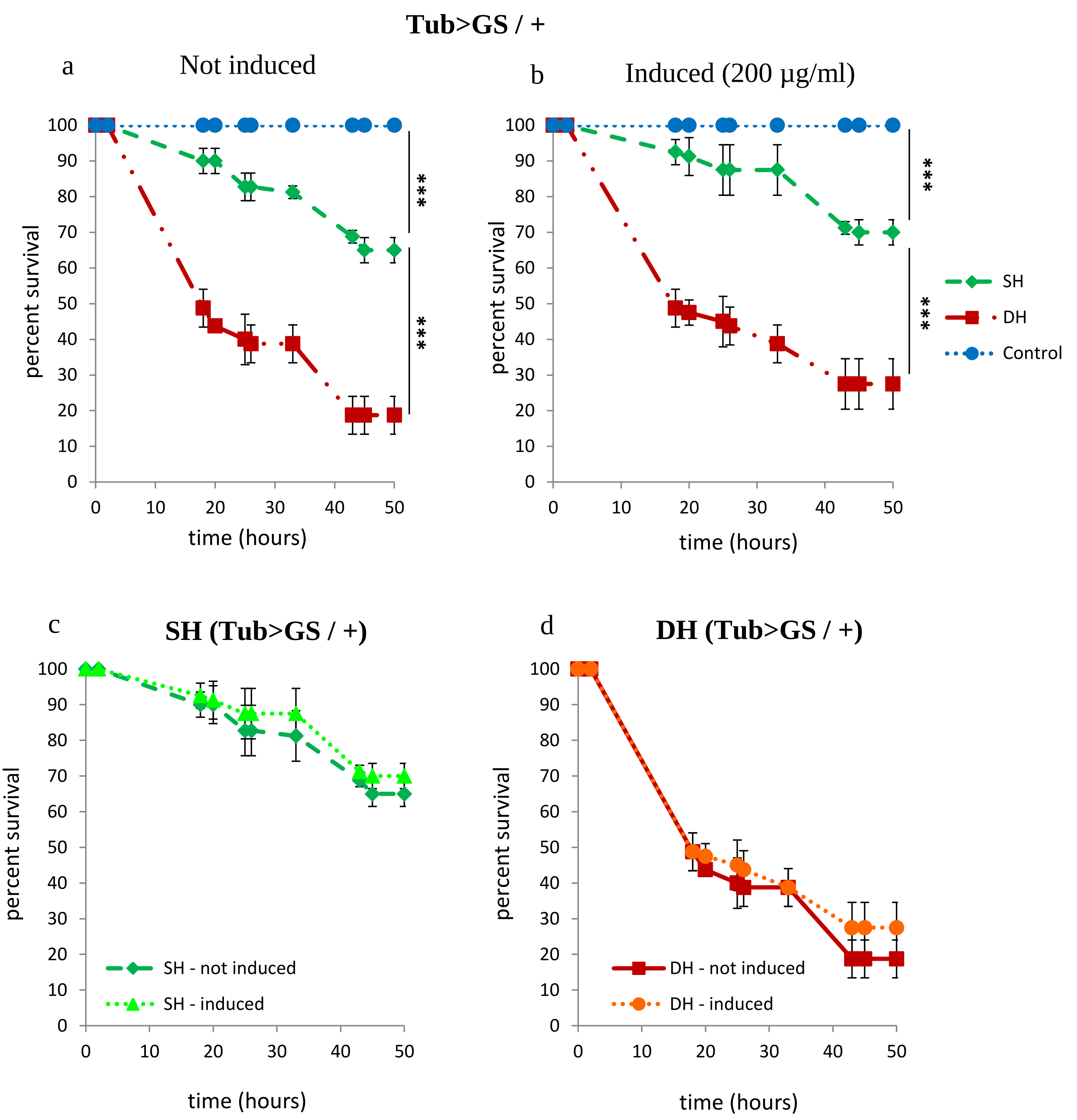

Tub>GS / +
a
Not induced
b
Induced (200 µg/ml)
percent survival
time (hours)
***
***
SH
DH
Control
c
SH (Tub>GS / +)
SH - not induced
SH - induced
d
DH (Tub>GS / +)
DH - not induced
DH - induced

Supplementary Figure S3: **RU486 (Mifepristone) does not affect the survival rate of SH, DH and control flies.** Sterile wounding or/and *Pe* infection were performed on Tubulin>GeneSwitch /+ adult females of 1 week old in absence (a) or in presence (b) of RU486 at indicated concentrations. Survival rates were determined up to 50 hours after treatment. (c, d) Same experiments as in a and b: (c) Survival rates of SH flies submitted (triangles) or not (lozenges) to RU486 feeding. $P = 0.82$; (d) Survival rates of DH flies submitted (circles) or not (squares) to RU486 feeding. $P = 0.93$. Each graph represents the observation of 80 flies among 2 different experiments (40 flies/ group/experiment). Statistical analyses were performed using the Log Rank test method (*** $P < 0.001$). Means of survival are shown, and error bars represent standard deviations.

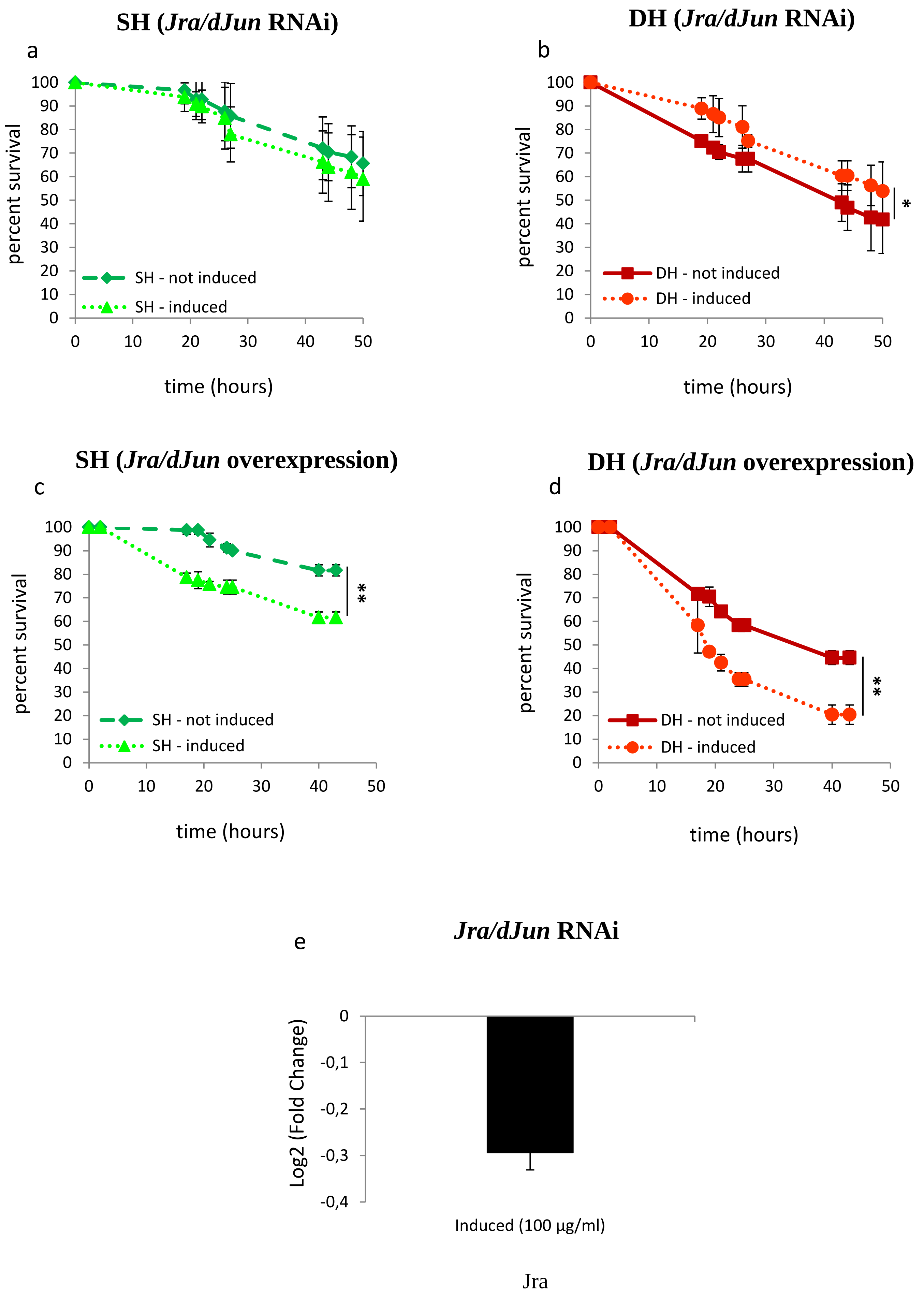

SH (Jra/dJun RNAi)
a
percent survival
time (hours)
SH - not induced
SH - induced
DH (Jra/dJun RNAi)
b
percent survival
time (hours)
DH - not induced
DH - induced
*
SH (Jra/dJun overexpression)
c
percent survival
time (hours)
SH - not induced
SH - induced
**
DH (Jra/dJun overexpression)
d
percent survival
time (hours)
DH - not induced
DH - induced
**
e
Jra/dJun RNAi
Log2 (Fold Change)
0
-0,1
-0,2
-0,3
-0,4
Induced (100 µg/ml)
Jra

Supplementary Figure S4: **Comparison of survival rates of SH (a, c) and DH (b, d) flies upon Jra/dJun loss and gain of function.** Experiments as in Figure 3. (a, b) Survival rates of SH (a) and DH (b) flies submitted (SH: triangles; DH: circles) or not (SH: lozenges; DH: squares) to *Jra/dJun* RNAi mediated inhibition. *Jra/dJun* loss of function did not affect the survival of SH flies (a, $P = 0.49$) but improved that of DH flies (b; $P = 0.03$). (c, d) Survival rates of SH (c) and DH (d) flies submitted (SH: triangles; DH: circles) or not (SH: lozenges; DH: squares) to *Jra/dJun* overexpression. *Jra/dJun* gain of function worsened the survival of both SH flies (c; $P = 0.0036$) and DH ones (d; $P = 0.0057$). Each group consisted of 120 flies observed among 3 different experiments (40 flies/ group /experiment). P values were calculated using the Log Rank test method (* $P < 0.05$; ** $P < 0.01$). Means of survival are shown, and error bars represent the standard deviation. (e) **Validation of Jra/dJun RNAi efficiency using RT-qPCR.** Black bar represent mean value of log2 (fold change) observed for the induced condition (presence of RU486 at indicated concentration) normalized to the "not induced condition (absence of RU486). P value assessing the difference in expression level between the induced and the not induced conditions was calculated using the T-Test method ($P = 0.034$).

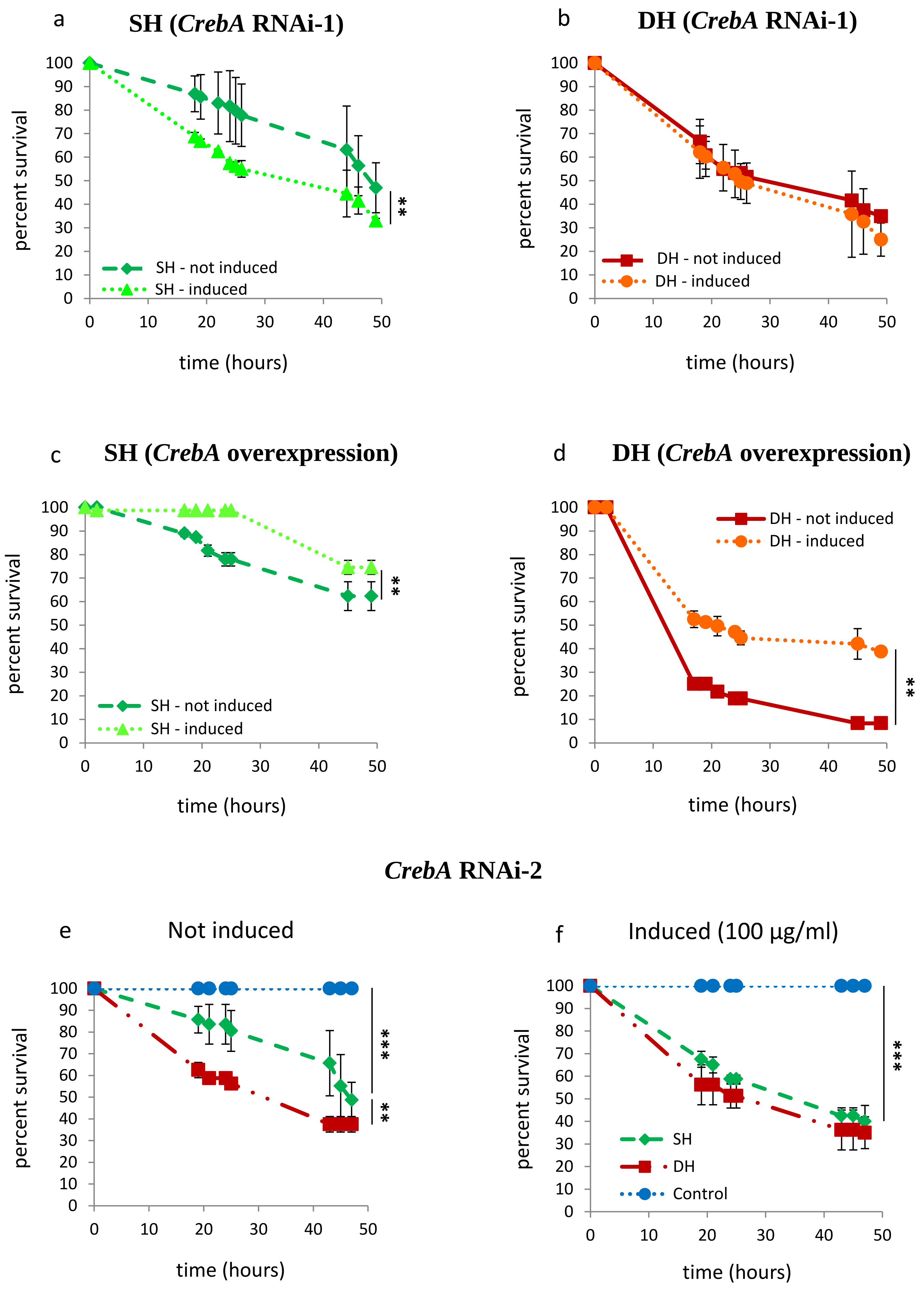

a
SH (CrebA RNAi-1)
percent survival
time (hours)
SH - not induced
SH - induced
**
b
DH (CrebA RNAi-1)
percent survival
time (hours)
DH - not induced
DH - induced
c
SH (CrebA overexpression)
percent survival
time (hours)
SH - not induced
SH - induced
**
d
DH (CrebA overexpression)
percent survival
time (hours)
DH - not induced
DH - induced
**
CrebA RNAi-2
e
Not induced
percent survival
time (hours)
***
**
f
Induced (100 µg/ml)
percent survival
time (hours)
SH
DH
Control
***

Supplementary Figure S5: **Comparison of survival rates of SH and DH flies upon CrebA loss and gain of function.** Experiments as in Figure 4. (a, b) Survival rates of SH (a) and DH (b) flies submitted (SH: triangles; DH: circles) or not (SH: lozenges; DH: squares) to RNAi mediated *CrebA* loss-of-function (CrebA RNAi-1). *CrebA* loss of function worsened the survival of SH flies (*P* = 0.0072) but did not impact the survival of DH flies (*P* = 0.89). (c, d) Survival rates of SH (c) and DH (d) flies submitted (SH: triangles; DH: circles) or not (SH: lozenges; DH: squares) to a *CrebA* over-expression. *CrebA* gain of function improved the survival of both SH and DH flies (*P* = 0.0053 and *P* = 0.0022 respectively). Each group consisted of 120 flies observed among 3 different experiments (40 flies / group / experiment). P values were calculated using the Log Rank test method (** *P* < 0.01; *** *P* < 0.001). Means of survival are shown and error bars represent the standard deviation. (e, f) Sterile wounding and/or *Pe* infection were performed on 1 week old adult females of Tubulin>GeneSwith /+; UAS-CrebA RNAi/+ genotype (CrebA RNAi-2) in the absence (e) or in the presence (f) of RU486 at indicated concentrations. Fly survival was observed up to 50 hours after treatment. Each group consisted of 120 flies observed among 3 different experiments (40 flies/ group/ experiment). P values were calculated using the Log Rank test method (** *P* < 0.01; *** *P* < 0.001).

Supplementary text T1: Detailed functional annotations of gene expression clusters.

Immediate early response to trauma and infection:

The 645 genes differentially expressed between SH and DH flies at 30 minutes, corresponding to genes of the 'immediate early' response to the challenges, were partitioned into 4 clusters.

- Cluster 1 contained genes highly expressed in DH flies compared to both SH and control ones (Fig. 2a). This gene set was enriched in GO annotation associated with 'response to stress' ($P = 2.10e^{-3}$) (Table 1). As detailed in the main text, several are known actors of the JNK signaling pathway (*Gadd45, Hsromega* and *Cabut*) while a number of genes participating in the heat shock response were also retrieved (*Hsp70Ab*, *Hsp70Bb*, *Hsp70Aa*, *Hsp70Ba*, *Hsp70Bc, Hsp70Bbb*).

- Cluster 2, which corresponded to genes showing higher expression in SH fly population compared to DH and control, was enriched in genes involved in the anti-bacterial humoral response ($P = 2.79e^{-5}$) (Table 1, see main text for detailed description of this cluster).

- Cluster 3 was composed of 84 genes down-regulated in DH flies compared to SH and control populations. This cluster was enriched in genes annotated as participating in eggshell formation ($P = 10^{-7}$) (Table 1), what may support a stress induced down-regulation of the reproductive status of DH females compared to both SH and control ones. Noticeably, i-*cis*Target analysis indicated that non-coding sequences of genes in cluster 3 were enriched for both Tata Binding Protein (TBP) and TBP associated factor 1 (Taf1) binding motifs, which are hallmarks of actively transcribed genes. This suggests that this cluster was comprised of genes that are usually actively transcribed by RNA polymerase II and may therefore be actively down-regulated upon combined trauma and infection.

The 147 genes of cluster 4 had a reduced expression in SH flies compared mainly to control ones and were enriched in GO annotation corresponding to ‘tissue development’ ($P = 0.03$) (Table 1). As in cluster 1, i-*cis*Target method retrieved an enrichment for Heat shock factor (Hsf) protein binding motif in the non-coding region of this gene set suggesting that a part of them are transcribed downstream this stress pathway. In addition, binding motif for the zinc finger TF ken and barbie (Ken) - a potential modulator of the JAK/STAT pathway[1] - was enriched in the non- coding sequences of cluster 4 genes. Finally, i-*cis*Target also retrieved the Blimp-1 protein binding motif. Blimp-1 is a transcriptional repressor recently identified as involved in precise timing of gene expression during Drosophila pupation[2]. It could therefore also be implicated in the controlled expression of these immediate early genes, which displayed dynamic expression within the first 30 minutes of the experiment.

Early response to trauma and infection:

At 3h, 786 genes were found to be differentially expressed and their expression profiles are divided into 3 clusters (Fig. 2b).

- Cluster 5 was composed of 355 genes down-regulated in SH flies compared to both DH and control ones. The GO enrichment analysis revealed the term 'response to stress' as being enriched ($P = 0.02$) (Table 1). A detailed description of this cluster is provided in the main text.

- Cluster 6 consisted of 416 up-regulated genes in the SH group, for which the GO enriched terms were ‘egg coat formation’ ($P = 4.56e^{-7}$), ‘transmembrane transport’ ($P = 2.41e^{-6}$) and ’immune response’ ($P = 0.03$) (Table 1). Genes involved in the formation of protective coverings of the egg thus appeared to be down-regulated in DH and control compared to SH flies. This may highlight a down-regulation of egg laying in response to trauma in DH flies, a (persistent) effect that has already been found after 30 minutes (cluster 3, Table 1). It is interesting to note that expression of innate immune response genes was maintained at higher levels in SH flies compared to DH ones, similarly to what was observed at 30 minutes (cluster 2, Table 1, see also main text). Importantly, a significant proportion of cluster 6 genes were identified as potential transcriptional targets of the CrebA/Creb3-like protein (hypergeometric test; $P = 4.90e^{-13}$) (see supplementary Table S5) and relaxing the stringency of the differential expression analysis by considering genes identified only by EdgeR, pointed out the *CrebA* gene as down-regulated in DH flies compared to SH ones (see Supplementary Table S2 and main text).

- Cluster 7 was composed of 15 genes up-regulated in DH flies, which did not exhibit statistically significant GO term enrichment.

Late phase of the early response to trauma and infection :

Finally, at 6h, 449 genes were found to be differentially expressed between the three conditions and were partitioned into 3 clusters (Fig. 2c).

- Cluster 8 was composed of 160 genes down-regulated in SH condition compared to both DH and controls. The GO enrichment analysis revealed the term ‘defense response’ ($P = 2.27e^{-4}$) suggesting that at 6 hours, genes linked to the innate immune response start to be activated in DH flies. This hypothesis was further supported by i-*cis*Target analysis, which highlighted the Relish binding motif enrichment in the potential regulatory regions of this gene set. Since this cluster comprised genes that are up-regulated in DH flies but also in control flies that were not subjected to bacterial challenge, this may reflect a fly response to inflammation. The 158 genes forming the cluster 9 were up-regulated in SH condition. Similarly to what was

observed at 3 hours (cluster 6), the GO enrichment analysis pointed out the term ‘egg coat formation’ (P = $4.05e^{-13}$). Cluster 10, which were composed of 131 up-regulated genes in both SH and DH flies compared to control alone, were significantly enriched in GO term associated to process of cell-cell signaling (P = $7.82e^{-7}$).

References

1. Arbouzova, N. I., Bach, E. A., & Zeidler, M. P. Ken & barbie selectively regulates the expression of a subset of Jak/STAT pathway target genes. *Curr Biol.* **16(1),** 80-8 (2006).

2. Akagi, K. *et al.* A biological timer in the fat body comprising Blimp-1, βFtz-f1 and Shade regulates pupation timing in Drosophila melanogaster. *Development.* **143(13),** 2410-6 (2016).
